# Infant gut microbiota and environment associate with juvenile idiopathic arthritis many years prior to disease onset, especially in genetically vulnerable children

**DOI:** 10.1016/j.ebiom.2023.104654

**Published:** 2023-06-15

**Authors:** Erik Kindgren, Angelica P. Ahrens, Eric W. Triplett, Johnny Ludvigsson

**Affiliations:** a Department of Pediatrics, Region Västra Götaland, Skaraborg Hospital, Skövde, Sweden; b Department of Biomedical and Clinical Sciences, Linköping University, Linköping, Sweden; c Department of Microbiology and Cell Science, Institute of Food and Agricultural Sciences, University of Florida, Gainesville, FL 32611-0700, USA; d Crown Princess Victoria’s Children’s Hospital, Region Östergötland, Linköping, SE 58185, Sweden

**Keywords:** Autoimmunity, Autoinflammatory disease, General population birth cohort, HLA, Antibiotics, Oligoarthritis, Pediatrics, Major histocompatibility complex (MHC), Prenatal, Microbiome

## Abstract

**Background:**

The etiology of juvenile idiopathic arthritis (JIA) is poorly understood. This study investigated genetic and environmental factors and infant gut microbiota in a prospective birth cohort to assess disease risk.

**Methods:**

Data was collected from the All Babies in Southeast Sweden (ABIS) population-based cohort (n = 17,055), 111 of whom later acquired JIA (ABIS_JIA_). Stool samples were collected at one year of age for 10.4%. To determine disease association, 16S rRNA gene sequences were analyzed, with and without confound adjustment. Genetic and environmental risks were assessed.

**Findings:**

ABIS_JIA_ had higher abundance of *Acidaminococcales, Prevotella 9*, and *Veillonella parvula* and lower abundance of *Coprococcus*, *Subdoligranulum*, *Phascolarctobacterium*, *Dialister* spp., *Bifidobacterium breve*, *Fusicatenibacter saccharivorans*, *Roseburia intestinalis*, and *Akkermansia muciniphila* (q’s < 0.05). *Parabacteroides distasonis* greatly increased the odds of later contracting JIA (OR = 6.7; 1.81–24.84, p = 0.0045). Shorter breastfeeding duration and increased antibiotic exposure compounded risk in a dose-dependent manner, especially in those with genetic predisposition.

**Interpretation:**

Microbial dysregulation in infancy may trigger or accelerate JIA development. Environmental risk factors have a stronger impact on genetically predisposed children. This study is the first to implicate microbial dysregulation in JIA at such an early age, with many bacterial taxa associated with risk factors. These findings provide opportunities for intervention or early screening and offer new insights into JIA pathogenesis.

**Funding:**

10.13039/501100004973Barndiabetesfonden; 10.13039/501100001861Swedish Council for Working Life and Social Research; 10.13039/501100004359Swedish Research Council; Östgöta Brandstodsbolag; 10.13039/501100000265Medical Research Council of Southeast Sweden; JDRF-Wallenberg Foundation; Linköping.

**Research in context**

**Evidence before this study**

Juvenile Idiopathic Arthritis (JIA) is a very debilitating autoimmune disease. Environmental factors are thought to play a significant role, but the etiology is complex and largely unknown. We searched Google Scholar and PubMed using the search terms “juvenile idiopathic arthritis microbiome”; “microbiome autoimmune disease”; “risk factors, juvenile idiopathic arthritis”; “genetics, juvenile idiopathic arthritis”; and “juvenile idiopathic arthritis etiology”. We included articles published in English between January 1, 2000, and December 30, 2021. Reference lists of these papers were used to identify other relevant literature. Various early life factors, like birth mode, diet, and antibiotic exposure, all of which can influence the intestinal microbiome, have been implicated in this autoimmune disease. Cross-sectional studies have found that children with JIA are known to have intestinal pathologies, including altered immunity, lesions, and hyperplasia, as well as an aberrant microbiome. Microbiome characteristics preceding disease in these children are not known.

**Added value of this study**

There are no extant studies of the gut microbiota in children in the years preceding a diagnosis. Here, using a prospective, birth cohort from Sweden ("All Babies in Southeast Sweden", or ABIS), we investigated whether gut microbiome signatures differed in infants who later acquired the disease. This is the first study, to our knowledge, to demonstrate that the gut microbiome differs in those with future JIA disease as early as in infancy. We also show that environmental and nutritional risk factors have a profound influence on disease risk in children with HLA haplotypes that are known to place a child at risk for autoimmune disease. We demonstrate how these risk factors associate with the bacteria most strongly implicated in future disease risk.

**Implications of all the available evidence**

Our findings suggest that microbial dysbiosis could emerge as early as in infancy in children with future disease, supporting a role of the microbiome in the preclinical stages. Bacterial strains more abundant in children with future disease include those previously connected to pro-inflammatory activation and/or intestinal barrier dysfunction. Bacterial strains that we found to be more abundant or prevalent in infants without future disease included butyrate producers and promoters of a healthy gut lining. The functional contributions of this dysbiosis should be explored in the context of mediating factors and especially in genetically vulnerable children.

## Introduction

Juvenile Idiopathic Arthritis (JIA) is a debilitating autoimmune disease,^1^ with environmental factors playing a significant role.^2^ Caesarean section (CS) is linked to autoimmune dysfunction, including JIA.^3^ Longer durations of breastfeeding seem protective.^4^^,^^5^ JIA risk increases in a dose-dependent manner with antibiotic exposure before two years of age, even after adjustment for infections.^6^ Evidence suggests that the greater the disruption by antibiotics, the greater the risk for JIA.^7^^,^^8^ The effect is stronger for antibiotics targeting anaerobes, likely due to longer impacts on the microbiota.^7^ Diet can also increase risk.^9^ These and other environmental factors influence the microbiome,^10^ which affects metabolic and immunological homeostasis, maintains the integrity of the intestinal mucosa, and trains the immune system. Even a short-term effect on the newborn’s intestinal microbiome can have long-term effects due to the early maturation of the immune system.

Intestinal mucosa dysfunction in children with JIA includes increased leakage of the intestinal epithelial barrier and signs of altered mucosal immunity, e.g., inflammatory lesions of the gut, ileal lymphonodular hyperplasia, and expression of human leukocyte antigen (HLA) -DR isotype (HLA-DR) at abnormal sites in the intestinal mucosa.^11^^,^^12^ Given that stool collection occurred in all existing gut microbiome JIA studies^13^, ^14^, ^15^, ^16^, ^17^, ^18^, ^19^ at the onset of disease or after several years of illness and treatment, separating the impact of the disease itself vs. treatment is problematic.

Several cross-sectional studies have shown differences in the gut microbiota of children with JIA. Study of children with new-onset JIA (mean age, 6.2 years) showed lower levels of *Firmicutes* and higher levels of *Bacteroidetes*, along with *Actinobacteria* and *Fusobacteria* species that were unique to JIA.^13^ Other cross-sectional studies have shown increased *Ruminococcaceae*,^15^ *Bacteroides*, *Enterococcus*, and *Klebsiella*,^14^ and decreased *Prevotella* species^14^ and *Clostridiaceae* and *Peptostreptococcaceae* in enthesitis-related arthritis (JIA-ERA).^15^ Increased *Ruminococcaceae* and decreased *Faecalibacterium* were also observed in non-enthesitis-related arthritis (JIA-nERA).^15^ Another study found a decrease in *Faecalibacterium prausnitzii* in treatment-naïve ERA patients, also seen in adults with longstanding spondyloarthritis (SpA), and increased *Bacteroides fragilis*, as well as reduced butanoate pathway capabilities by shotgun metagenomics.^18^ In a study of 109 treatment-naive JIA patients with <6 months of disease and 107 geographically matched healthy children, *Erysipelotrichaceae* and *Faecalibacterium prausnitzii* were increased in JIA patients, which differed from other studies.^17^ Overall, these cross-sectional studies suggest that differences in gut microbes may play a role in triggering pro-inflammatory reactions, potentially contributing to the pathogenesis of JIA. It is also possible that these differences in gut microbes may impact the development of the immune system. However, it should be noted that all of these studies were conducted after the development of the disease.

There are no extant studies of the gut microbiota in children in the years preceding a diagnosis. Here, a novel question is explored. Does the gut microbiome differ in those with future JIA disease as early as in infancy? We also assessed the effects of environmental and nutritional risk factors, separately and cumulatively, and in combination with HLA haplotypes that are known to place a child at risk.

## Methods

### Study design and participants

Parents of all children born from October 1, 1997 to October 1, 1999 in southeastern Sweden were asked to participate in All Babies in Southeast Sweden (ABIS). The primary purpose was to investigate environmental and genetic factors on immune-mediated diseases. Of the 21,700 families invited, 17,055 agreed (78.6%). Questionnaires including nutrition, infections, and drug use were answered at birth and at recurring intervals during childhood. Missing questionnaire data was identified at random. Some analyses were based on questionnaires at birth with answers available from 14,482 to 16,489 questionnaires, while other analyses are based on the questionnaire at one year of age of the child, with answers available from 10,057 to 10,168 questionnaires.

**Fig. 1:**
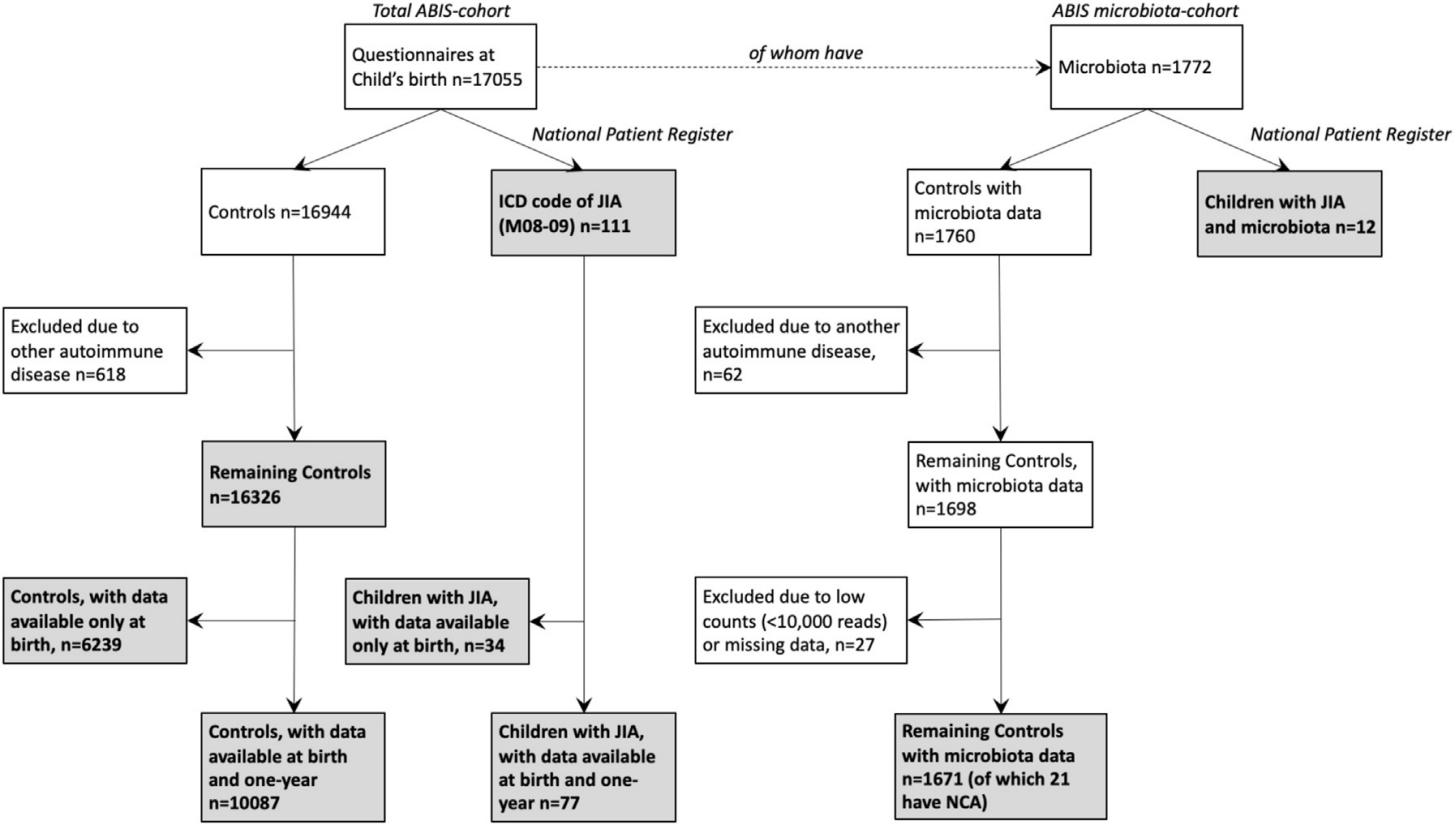
**The All Babies in Southeast Sweden (ABIS) study sample.** Filtering of the ABIS children is depicted. A total of 111 ABIS children were later diagnosed with Juvenile Idiopathic Arthritis (JIA), ABIS_JIA_. Diagnoses were obtained from the Swedish National Patient Register through 31 December 2020. Other autoimmune disease includes Celiac disease, inflammatory bowel disease, hypothyroidism and type 1 diabetes. ICD = International classification of diseases, NCA = non-chronic arthritis.

Diagnoses are obtained using the Swedish National Patient Register, which serves as a repository of specialist consultations in outpatient care from public and private care providers. The Swedish Pediatric Rheumatology Registry provided further verification and JIA subclassifications. A total of 111 children received a qualifying JIA diagnosis by 31st of December 2020 (ABIS_JIA_), defined by an International Classification of Diseases (ICD) code for JIA (M08-09) on at least two occasions, at least six months apart (Fig. 1). These criteria were instated to reduce the likelihood of false positive JIA diagnoses. Patients with misdiagnoses were excluded. JIA diagnoses occurred at 11.1 ± 5.5 years of age, with the earliest diagnosis at 2 years. Cohort characteristics are in Table 1, with Mann–Whitney or χ^2^. The gestational age of the controls was 39.7 ± 2.4 weeks and 39.5 ± 2.0 weeks for the JIA group.

**Table 1:** Basic characteristics of the study population.

	The ABIS cohort		p^a^	ABIS with microbiome		p^b^	p^c^
	Controls	ABIS_JIA_		Controls	ABIS_JIA_		
**Questionnaires at child’s birth, *n***	16,944	111		1671	12		
**Sex**							
Female	8082 (47.7%)	73 (66.4%)	<0.001	802 (48%)	8 (67%)	0.195	0.876
Male	8862 (52.3%)	37 (33.6%)		869 (52%)	4 (33%)		
**Gestational age, weeks**	39.7 (2.4)	39.5 (2.0)	0.253	39.7 (1.8)	39.8 (1.1)	0.887	0.596
**Birth weight, g**	3579 (560)	3454 (605)	0.065	3587 (540)	3537 (494)	0.931	0.572
**Birth length, cm**	51 (2)	50 (3)	0.031	51 (2)	50 (2)	0.243	0.108
**Age at diagnosis, years**	–	11.1 (5.5)		–	13.0 (5.3)		0.180
**Family history/Heredity for JIA/RA**							
Mother with RA	150 (1%)	2 (2%)	0.366	15 (1%)	1 (8%)	0.014	<0.001
Father with RA	63 (0%)	0 (0%)	0.503	2 (0%)	0 (0%)	0.899	0.189
Siblings with JIA/RA	46 (0%)	0 (0%)	0.567	5 (0%)	0 (0%)	0.841	0.989
2nd degree family member with RA	1359 (9%)	18 (17%)	0.005	140 (9%)	1 (8%)	0.958	0.297
**Age mother when the child was born, years**	30 (5)	28 (5)	0.008	30 (4)	29 (5)	0.144	0.078
**Age father when the child was born, years**	32 (5)	31 (5)	0.066	32 (5)	31 (5)	0.344	0.108
**Origin parents**							
Both parents born in Sweden	13,605 (89%)	103 (95%)	0.036	1369 (91%)	11 (92%)	0.912	0.184
Both parents born outside Sweden	479 (3%)	1 (1%)	0.188	30 (2%)	1 (8%)	0.618	0.017
**Education mother at child’s birth**							
Elementary school 9 years	1316 (9%)	6 (6%)	0.147	79 (5%)	0 (0%)	0.126	0.141
High school	9089 (60%)	72 (66%)	0.311	861 (57%)	9 (75%)	0.091	0.872
College	4867 (32%)	31 (28%)	0.216	578 (38%)	3 (25%)	0.587	0.677
**Education father at child’s birth**							
Elementary school 9 years	2039 (14%)	14 (13%)	0.801	161 (11%)	2 (17%)	0.882	0.411
High school	9310 (62%)	77 (71%)	0.114	928 (62%)	9 (73%)	0.111	0.298
College	3697 (25%)	18 (17%)	0.089	402 (27%)	1 (8%)	0.351	0.436
**Living conditions (age 1 yr)**							
House	8778 (57%)	59 (55%)	0.761	928 (61%)	8 (67%)	0.788	0.757
Apartment	5989 (39%)	42 (39%)	0.812	542 (36%)	4 (33%)	0.592	0.892
Other	508 (3%)	6 (6%)	0.071	43 (3%)	0 (0%)	0.322	0.333
Solitary in the countryside	2124 (14%)	15 (14%)	0.762	206 (14%)	2 (25%)	0.221	0.114
<500 inhabitants	1336 (9%)	15 (14%)	0.546	140 (9%)	2 (13%)	0.633	0.873
500–3000 inhabitants	3436 (23%)	21 (19%)	0.211	320 (21%)	2 (13%)	0.341	0.101
Larger town	8119 (54%)	57 (53%)	0.673	826 (55%)	6 (50%)	0.602	0.647
**Questionnaires at 1 year, *n***	10,087	77		1671	12		
Weight at 1 year, kg	10.1 (1.2)	9.9 (1.2)	0.173	10.1 (1.2)	9.7 (0.7)	0.253	0.274
Height at 1 year, cm	76 (3)	75 (3)	0.123	76 (3)	76 (3)	0.572	0.116
Body-mass index at 1 year, kg/m^2^	17.4 (1.5)	17.4 (1.6)	0.653	17.4 (1.5)	17.0 (1.0)	0.386	0.744

Data are n (%), median (IQR), or mean (SD). P-value from Mann-Whitney’s U-test or χ^2^. JIA = juvenile idiopathic arthritis.

a,b Comparison between cases and controls.

c Comparison between the entire ABIS-cohort and the subgroup with stool samples.

Stool samples at one year of age were available in twelve ABIS_JIA_ (diagnosis at 13.8 ± 4.6 years, in every case after the age of three). There were no differences in gestational age, birth weight, age of diagnosis, or family history of JIA in those with microbiome data and the rest of ABIS (Table 1).

### Procedures

#### Genotyping

HLA typing was performed on 3623 children (ABIS_JIA_, n = 37). HLA-DR and -DQ genotypes were determined using sequence-specific hybridization with lanthanide-labelled oligonucleotide probes.^20^

#### Microbiome profiling

Parents of 1772 individuals submitted stool samples from their child at one year. Samples were collected from the child’s diaper using a sterile spatula and tubes provided by WellBaby Clinic. The samples were frozen rapidly after collection, in the infant’s home or clinic, and then sent rapidly to Clinical Experimental Research Centre, where they were stored dry at −80 °C.

DNA was extracted using the E.Z.N.A. Stool Extraction Kit according to the manufacturer’s protocol (Omega Biotech, Doraville, CA). Samples were randomized to prevent the introduction of bias during extraction, and blank negative controls were used to rule out contamination. DNA was quantified and evaluated for purity using a Nanodrop spectrophotometer (Thermo Scientific, Wilmington, DE). Sample-specific bar-coded primers 341F and 806R, targeting the V3–V4 variable regions of the 16S rRNA gene, were used, and PCR amplification was performed. Final libraries were quantified and pooled to equimolar amounts. Sequencing was performed across ten separate runs, in parallel with the entire bacterial genome in a MiSeq instrument (Illumina Inc., San Diego, CA, USA) with 2 × 300 bp readings and V3 chemistry at the Interdisciplinary Center for Biotechnology Research, University of Florida, Gainesville, Florida, USA.

Sequences were merged and demultiplexed in Qiime1^21^ using the join_paired_ends.py, split_libraries_fastq.py, and split_sequence_file_on_sample_ids.py functions. Amplicon sequencing variants (ASV) were derived using the DADA2 R package^22^ after removing low-quality reads and trimming sequences to 400 nucleotides. Each run was processed separately, and the sequence tables merged for chimera removal by the consensus method and taxonomic assignment by SILVA 138.1.^23^ Chimeras accounted for 0.7% of merged reads. After removal of samples with low read counts (<10,000 reads), 1743 samples remained for environmental analysis, with a total of 107,959,956 reads and 13,197 unique ASVs (see Supplemental Table S1 for full sequences). A median of 57,844 reads was observed (minimum of 1059 and maximum of 776,158), with an average of 61,939.2 reads per sample. A total of 1683 samples were included in differential abundance analysis, due to exclusion of samples from children who later acquired an autoimmune disease besides JIA.

### Analysis of environmental features

**Fig. 2:**
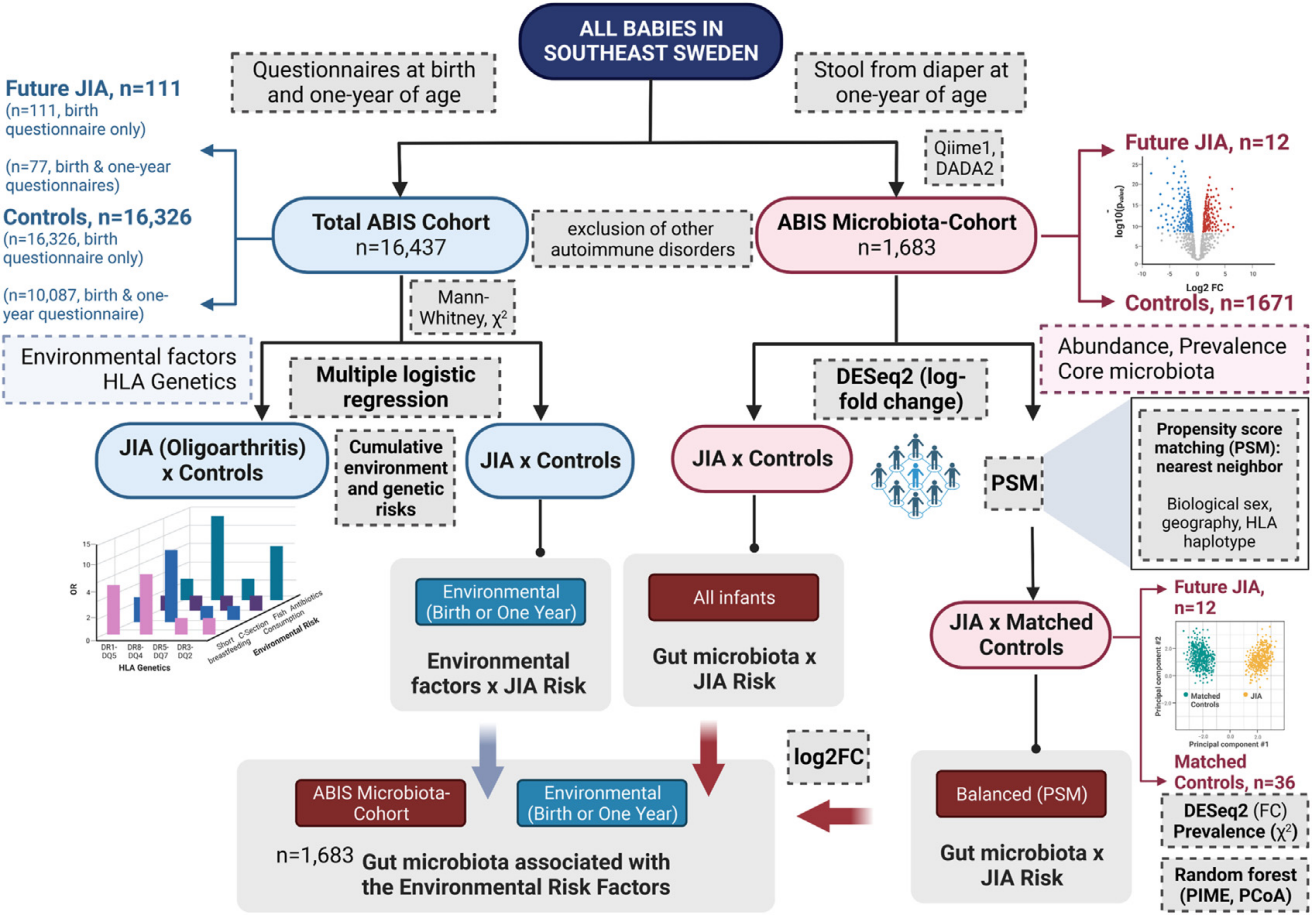
**Analytical workflow for the All Babies in Southeast Sweden (ABIS) JIA investigation.** Analysis of environmental and genetic factors in the total ABIS cohort (n = 16,437) with 111 ABIS children later diagnosed with Juvenile Idiopathic Arthritis (JIA) including single factor using Mann–Whitney and χ^2^ as well as cumulative environmental and genetic risk calculations using multiple logistic regression. Analysis of microbiome features in the ABIS Microbiota-Cohort (n = 1683 with n = 12 future JIA) and a subset of matched controls and (n = 36 vs. n = 12 with JIA), selected by propensity score matching (PSM). Microbiome analysis consisted of differential abundance (Differential gene expression analysis based on negative binomial distribution R package, DESeq2, with log-fold change), prevalence (χ^2^), and core microbiome communities (Prevalence Interval for Microbiome Evaluation R package, PIME, with random forest and principal components analysis). After identifying the top microbes that were most significantly differentially abundant or prevalent by these analyses, we assessed correlations with the environmental risk factors, using log2 fold change (FC) analysis in SPSS in the total ABIS microbiota-cohort (n = 1683).

Variance homogeneity was tested with the Levene test. Non-parametric tests were used for data that deviated from a Gaussian distribution. Multiple logistic regression was used to estimate odds ratios (OR). Analyses were performed using IBM SPSS Statistics, v28.0 (IBM Corp., Armonk, NY, USA). An analytical workflow for this investigation, from analysis to environmental features to microbiome communities and taxonomic abundances, is provided as Fig. 2.

### Analysis of microbiome features

To assess potential confounds on microbial diversity, ASVs were compositionally transformed with the microbiome R package.^24^ Distances were determined using the Bray–Curtis method in phyloseq and a permutational multivariate analysis of variance (PERMANOVA) with 1000 permutations conducted on ASVs using the vegan R package,^25^ excluding samples with missing data on HLA, sex, or geography (N = 1387). Subsequent confounds were accounted for by propensity score matching.

Differential abundance was detected using differential gene expression analysis based on the negative binomial distribution with the DESeq2 R package.^26^ This analysis was performed against ABIS_JIA_ (n = 12) and three separate sets of controls: n = 1671, all without JIA (excluding 62 infants with a future diagnosis of another autoimmune disease); n = 21, children with an episode of a transient non-chronic arthritis (NCA); and a subset of n = 30 controls, selected to balance the microbiome confounds. Estimates of logarithmic fold change (log2FC) and shrinkage of effect size were calculated using DESeq2, with false discovery rate (FDR) adjustments, after independent filtering based on normalized counts. The Wald test estimated the standard error of log2FC and Cook’s distance as a diagnostic for outliers.

To account for immune-mediating genetics and other confounders of microbial composition, a subset of controls was selected for the twelve JIA cases in a 3:1 ratio based on sex, geography, and distribution of the 17 distinct HLA haplotypes. For this, we used a propensity score, nearest neighbour matching (PSM) algorithm by logistic regression in the matchIt R package.^27^ This approach achieves balance in the covariates for causal inference. Cohort characteristics comparisons between control and future JIA groups, before and after PSM, are provided in Supplemental Table S2.

Community architecture was investigated within the matched cohort using the PIME R package,^28^ which filters bacteria using random forest at specified prevalence intervals. An out-of-bag (OOB) error rate of 0.0417 was observed at 65% prevalence, representing 27 genera across 2,105,070 reads. Principal components analysis was applied to the filtered taxa in R to distinguish the degree of separation in the core microbial communities between future cases and matched controls.

### Environmental factors and the microbiome

Differentially abundant taxa were subsequently tested against environmental features (antibiotic exposure, breastfeeding duration, fish consumption, siblings, and mode of delivery) using log2FC in SPSS, with an FDR of 10% by Benjamini–Hochberg and standard error calculated as (std error/mean)∗log2e. Environmental factors were dichotomized as: three or more antibiotic treatments across pregnancy and the first year of life, less than four months of exclusive or less than eight months of total breastfeeding, and consumption of fish more than once per week prenatally or in the first year of life.

### Ethics

The ABIS study has ethical approval from the Research Ethics Committees of the Faculty of Health Science at Linköping University, Sweden, Ref. 1997/96287 and 2003/03-092 and the Medical Faculty of Lund University, Sweden (Dnr 99227, Dnr 99321). All parents in ABIS provided their informed consent after careful oral and written information. Microbiome analyses were performed at the University of Florida after approval from the University of Florida’s Institutional Review Board as an exempt study assigned as IRB201800903.

### Role of the funding source

Study sponsors had no role in the collection, analysis, or interpretation of data, nor in writing the report or the decision to submit the paper for publication. Funding sources did not play a role in the writing of the manuscript or the decision to submit it for publication. Authors were not paid to write this article by a pharmaceutical company or other agency. Authors were not precluded from accessing data in the study, and they accept responsibility to submit for publication.

## Results

### Environmental and perinatal factors

**Fig. 3:**
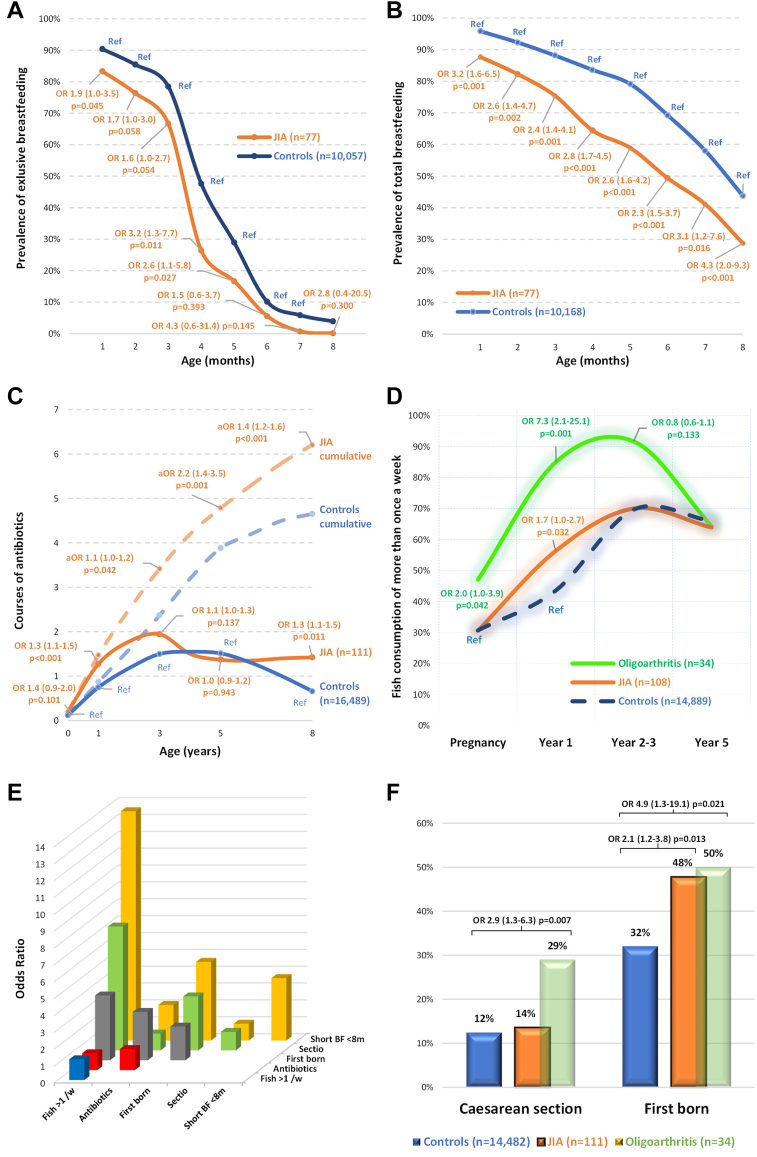
**Environmental factors and JIA.** OR = odds ratio. Compounded effects of the environmental factors. A) Prevalence of exclusive breastfeeding at one-month increments from one to eight or more months in infants with a future JIA diagnosis (n = 77) and controls (n = 10,057). B) Prevalence of total breastfeeding at one-month increments from one to eight or more months in infants with a future JIA diagnosis (n = 77) and controls (n = 10,168). C) Exposure to antibiotics prenatally and during childhood, by cumulative courses of antibiotics, across the first eight years of life, in infants with a future JIA diagnosis (n = 111) and controls (n = 16,489). D) Fish consumption by the mother during pregnancy and by the child at one, two to three, and five years of age, in infants with a future JIA diagnosis (n = 108), infants with a future oligoarthritis diagnosis (n = 34), and controls (n = 14,889). E) Compounded effects of the environmental factors, with ORs. F) Prevalence of Caesarean section and being a firstborn child, in infants with a future JIA diagnosis (n = 111), infants with a future oligoarthritis diagnosis (n = 34), and controls (n = 14,482).

JIA risk increased with exclusive breastfeeding for less than four months (Odds ratio, OR = 3.2; CI 1.3–7.7, p = 0.011; Fig. 3A) or total breastfeeding for less than eight months (OR = 4.3; CI 2.0–9.3, p < 0.001; Fig. 3B). Exclusive breastfeeding duration for controls and JIA were 4.5 ± 1.9 months and 3.7 ± 1.7 months, respectively (median 4 months for both). Total breastfeeding duration for controls and JIA were 7.1 ± 2.3 and 5.9 ± 2.9, respectively (median of 8 and 6.5). Mode of delivery did not differ between JIA and control groups, with 12% of the controls and 14% of ABIS_JIA_ having been delivered by CS (OR = 1.1; CI 0.6–1.8, p = 0.866; Fig. 3F). In a subgroup analysis, however, 29% of those who later developed oligoarticular JIA had been delivered by CS (OR = 2.9; CI 1.3–6.3, p = 0.007). Being a firstborn increased odds of JIA twofold (OR = 2.1; CI 1.2–3.8, p = 0.013; Fig. 3F) and of oligoarthritis fivefold (OR = 4.9; CI 1.3–19.1, p = 0.021). Fish consumption in the first year of life was associated with JIA (OR = 1.7 (1.0–2.7), p = 0.032) as well oligoarticular JIA (OR = 7.3; CI 2.1–25.1, p = 0.001; Fig. 3D) but not at years two to three or year five. Fish consumption of the mother during pregnancy was associated with oligarthritis in the child (OR = 2.0; CI 1.0–3.9, p = 0.042). Antibiotic exposure (maternal use during the fetal period or infant use in the first year) increased the odds of JIA (OR = 1.3; CI 1.1–1.5, p < 0.001, Fig. 3C). Over the first year of life, controls and children with future JIA had been exposed to 0.9 ± 1.2 and 1.5 ± 1.9 courses of antibiotics, respectively (median of 0 and 1 courses). Risk increased with the cumulative number of courses in the first five years especially (OR = 2.2; CI 1.4–3.5, p = 0.001).

Combinations of these early-life factors compounded JIA risk (Fig. 3E). Short-term total breastfeeding (fewer than eight months) and consumption of fish in the first year showed the greatest effect, with OR = 13.4 (4.6–39.1), p < 0.001.

### Genetic contributions

HLA-DR1-DQ5 was present in 35.1% of ABIS_JIA_ compared to 22.3% of controls (OR = 1.9; 1.0–3.7, p = 0.068), and homozygous DR1-DQ5 was more common in ABIS_JIA_, 8.1% vs. 2.4% (OR = 3.6; 1.1–12.1, p = 0.035). The association was stronger in those who exclusively breastfed for two months or less (n = 443), where 26% of controls and 67% of ABIS_JIA_ had DR1-DQ5 (OR = 6.1; 1.1–33.7, p = 0.038).

DR8-DQ4 was more common in ABIS_JIA_ (18.9% vs. 8.3%) with OR = 2.6 (1.1–5.9), p = 0.026, and overrepresented in the Oligoarthritis subgroup, where 41.7% had this allele (OR = 7.9; 2.5–24.9, p < 0.001). Homozygous DR8-DQ4 was very rare in the entire cohort (0.4%). More than half of ABIS_JIA_ and future Oligoarthritis (51.4% and 58.3%) had DR1-DQ5 and/or DR8-DQ4, compared to 29.6% of controls, with OR = 2.5 (1.3–4.8), p = 0.005 for JIA and OR = 3.3 (1.0–10.5), p = 0.041 for Oligoarthritis. Across those children delivered by CS (n = 384), DR8-DQ4 was more frequent in ABIS_JIA_ (33%) compared to controls (7%), with an OR = 7.1 (1.2–40.4, p = 0.028).

**Fig. 4:**
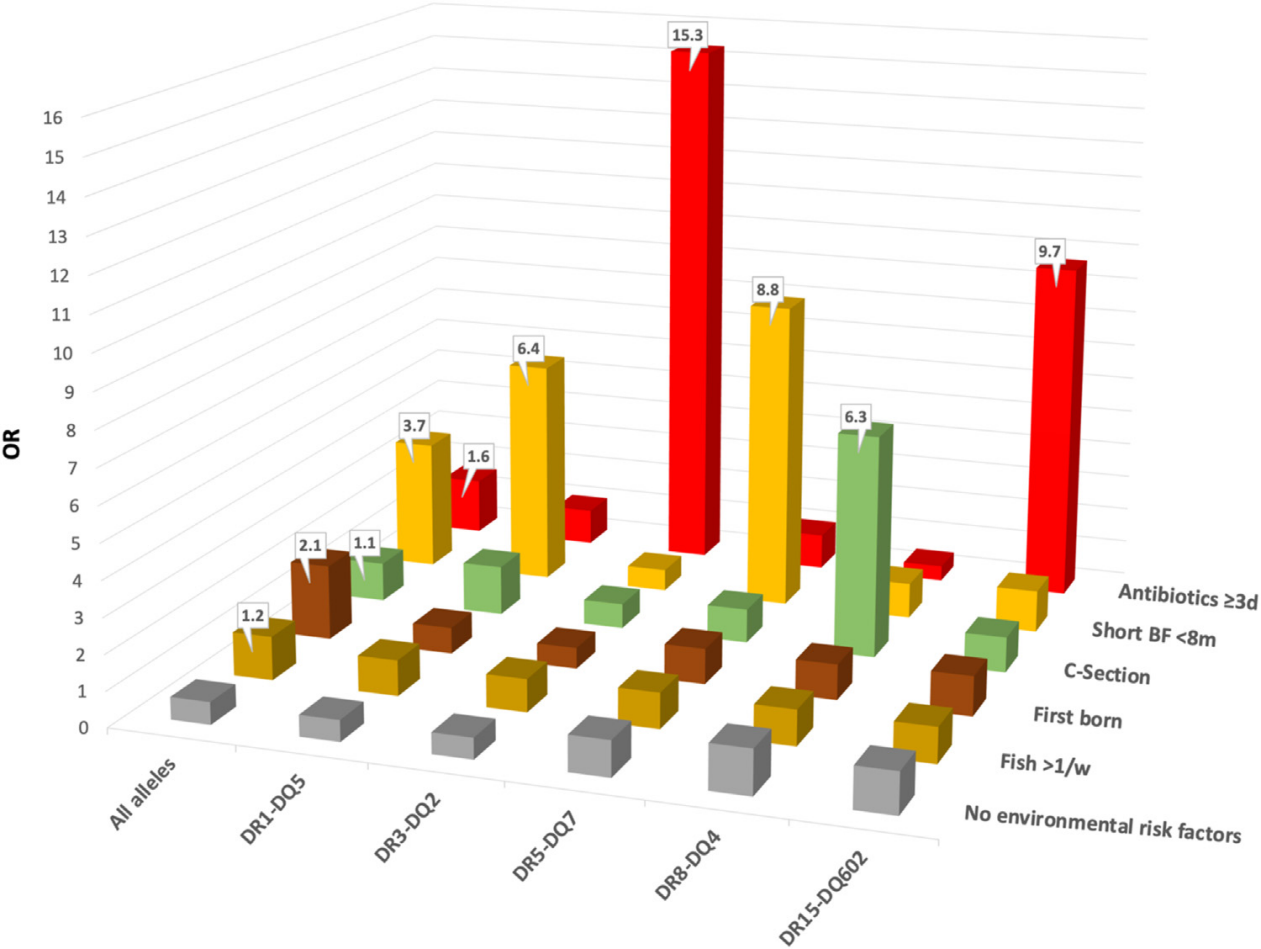
**Gene-environment interactions.** Odds ratios of developing Juvenile Idiopathic Arthritis (JIA) in subjects exposed to different combinations of environmental risk factors and HLA–DR genes compared with those not exposed to environmental factors.

Overall, exposure to *three* or more courses of antibiotics during pregnancy or the first year resulted in a 1.6 increased odds of developing JIA (OR = 1.1–2.4, p = 0.028). Children with DR3-DQ2 had a further increased risk for JIA (OR = 15.3; 3.2–74.5, p < 0.001), as were those with DR15-DQ602 (OR = 9.6; 1.9–50.2, p = 0.007) when exposed to three or more courses of antibiotics (Fig. 4).

Short-term total breastfeeding (fewer than eight months) involved an increased odds of JIA (3.7), with two alleles increasing the odds further; DR1-DQ5 (OR = 6.4; 1.2–35.3, p = 0.033) and DR5-DQ7 (OR = 8.8; 1.0–73.3, p = 0.045). CS delivery did not increase the risk of JIA (OR = 1.1; 0.6–2.0, p = 0.720); however, DR8-DQ4 in combination with CS did (OR = 7.5; 1.7–33.1, p = 0.007). The subgroup Oligoarthritis showed an association with CS (OR = 2.9; 1.3–6.3, p = 0.007), which was stronger in the presence of DR8-DQ4 (OR = 27.8; 5.7–135.5, p < 0.001).

### Microbiome differences in ABIS_JIA_

#### Microbiome diversity

Sex and geography contributed to Bray–Curtis microbial diversity (Supplemental Table S3). Three HLA haplotypes (DR13-DQ603, DR14-DQ5, and DR4-DQ7) were near significant (p’s < 0.1). Body mass index (BMI) at one-year was not associated (PERMANOVA, p = 0.68) at the ASV-level (n = 1314), nor was BMI at two years (n = 59, p = 0.23), five years (n = 65, p = 0.32) or eight years (n = 27, p = 0.30).

#### Differences across taxonomic ranks between controls and ABIS_JIA_

**Fig. 5:**
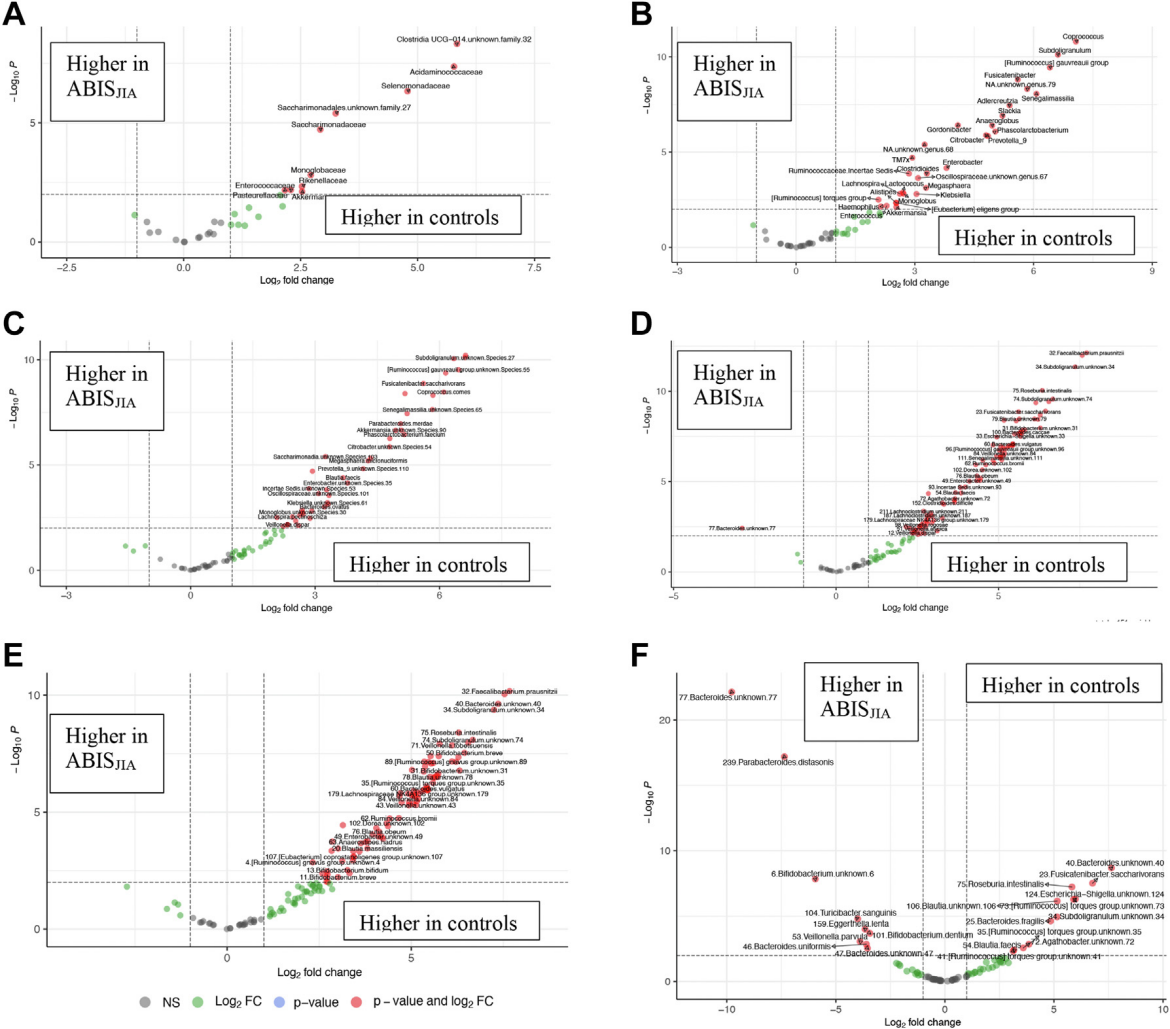
**Differentially abundant microbiota in infants with future JIA.** A–E) Comparisons of future JIA (N = 12), ABIS_JIA_, against all controls (N = 1671) in relative abundance. A total of 25 phyla, 32 families, 70 genera, 100 species, 153 ASVs were compared. See Supplemental Table S4 for full statistics. F) Comparisons against non-chronic arthritis controls (N = 21) at the ASV level. See Supplemental Table S5 for full statistics, including genus and species. Fold change was calculated using DESeq2 with false discovery rate correction. Thus, all p values reflect adjusted p values after FDR correction. Differentially abundant bacteria depicted had normalized base mean counts ≥30.

Many differences in relative abundance were observed between ABIS_JIA_ (N = 12) vs. controls (n = 1671) (Fig. 5A–E; Supplemental Table S4). *Verrucomicrobiales*, *Monoglobales*, *Saccharimonadales*, *Clostridia UCG-014*, and *Acidaminococcales* were higher in controls, as were *Coprococcus*, *Subdoligranulum*, *Fusicatenibacter*, *Phascolarctobacterium*, *Alistipes*, *Akkermansia*, *Monoglobus*, *Parasutterella*, *Tyzzerella*, *Enterobacter*, *Christensenellaceae* spp., *Lachnospira*, *Oscillibacter*, *Roseburia*, and others. Also reduced in ABIS_JIA_ were *Fusicatenibacter saccharivorans*, *Coprococcus comes*, *Blautia faecis*, *Lachnospira pectinoschiza*, *Adlercreutzia equoliaciens*, *Roseburia hominis*, *Blautia obeum*, *Bacteroides caccae*, *Bifidobacterium breve*, *Bacteroides ovatus*, and others. Four ASVs were more abundant in ABIS_JIA_, including ASV-239 *Parabacteroides distasonis*, ASV-77 *Bacteroides* sp., ASV-539 *Lachnoclostridium* sp., and ASV-365 *Bacteroides* sp.

#### Differences between NCA controls and ABIS_JIA_

Differences in relative abundance were also observed between ABIS_JIA_ and NCA controls (Supplemental Table S5). *Fusicatenibacter*, *Subdoligranulum*, *Senegalimassilia*, and *Coprococcus* were higher in NCA controls. Seven species with normalized base mean counts ≥30 were differentially abundant, including *Parabacteroides distasonis* (FC = −4.4, padj = 0.000026) and *Turicibacter sanguinis* (FC = −3.8, padj = 0.00024), which were higher in ABIS_JIA_, and *Fusicatenibacter saccharivorans* and *Roseburia intestinalis*, which were higher in controls. At the ASV level, 21 taxa with normalized base mean counts >30 were differentially abundant (Fig. 5F; Supplemental Table S5).

#### Microbiome differences after matching

*Parabacteroides distasonis* was seen in 75% of ABIS_JIA_ but only 25% of matched controls, χ^2^ = 9.6 (1, n = 48), p = 0.002, with 9.0-increased odds of future disease (1.99–40.69, p = 0.0043). It was only present in 23.8% of NCA controls (n = 21) and 30.9% of all controls (n = 1671). Across ABIS, the presence of *P. distasonis* was associated with 6.7× increased odds of later contracting JIA (1.81–24.84, p = 0.0045). Also, all but one of ABIS_JIA_ (91.7%) harbored an unknown species of *Enterococcus*, compared to 58.3% of matched controls (p = 0.034).

**Fig. 6:**
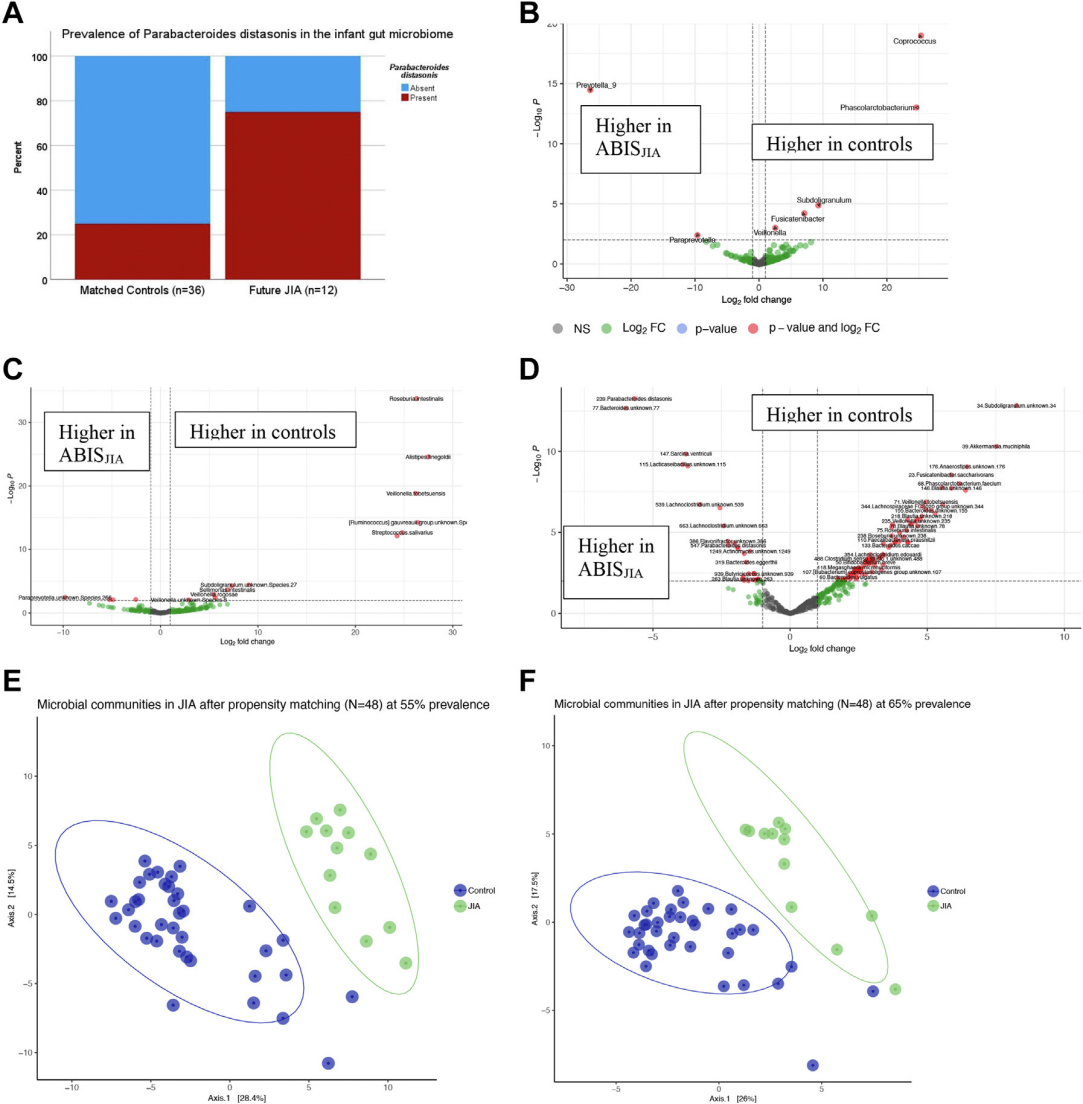
**Differentially abundant bacteria in future JIA after accounting for sex, geography, and HLA genetics.** A) Prevalence of *Parabacteroides distasonis* between the groups. Differences between future JIA, ABIS_JIA_ (N = 12) and selected controls (N = 36) are depicted at the B) genus, C) species, and D) ASV levels. A total of 186 genera, 190 species, and 149,512 ASVs were compared. Controls were selected using propensity score matching to achieve balance in the sex, geography, and the 17 HLA haplotypes in the cases and controls. Fold change analysis was carried out in DESeq2 with false discovery rate correction. Thus, all p values reflect FDR correction. Significant bacteria had normalized base mean counts >10. E and F) Community architecture of the infant microbiome across future JIA and matched controls. Principal components analysis of the species represented by the 55% prevalence threshold and of the genera represented by the 65% prevalence thresholds, respectively, generated using the PIME R package. At the 55% prevalence threshold of species, the out-of-bag (OOB error) rate classification was 2.08% and at the 65% threshold, OOB error was 4.17% (Supplemental Table S6).

*Fusicatenibacter* and *Subdoligranulum* were lower in ABIS_JIA_ (Fig. 6A). Notably, *Prevotella 9* was observed in 30% of ABIS_JIA_ and absent in every control in the matched subcohort (Fig. 6B). Higher in controls were *Sellimonas intestinalis*, *Roseburia intestinalis*, *Phascolarctobacterium faecium*, and *Fusicatenibacter saccharivorans*, among others (Fig. 6C). ASV-38 *Akkermansia muciniphila*, ASV-94 *Veillonella parvula*, ASV-32 *Faecalibacterium prausntzii*, and several strains of *Subdoligranulum*, *Blautia*, *Bacteroides*, *Ruminococcus*, and *Lachnoclostridium* were more abundant in the controls (Fig. 6D). Community differences were observed at the species (Fig. 6E) and genus level (Fig. 6F), with significant separation and an OOB-error rate of 2.08 and 4.17% at the 55% and 65% prevalence thresholds, respectively (Fig. 6F). The species and genera contributing most significantly to these classifications are listed in Supplemental Table S6.

### Environmental factors and microbiota

**Fig. 7:**
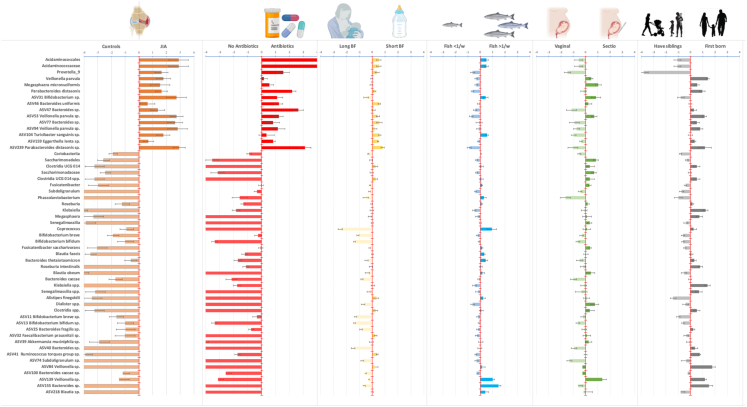
**Environmental associations of the top microbes most differentially abundant across future JIA and controls at one year of age.** Statistical significance from all analyses was evaluated with a false discovery rate of 10%, calculated using the Benjamini–Hochberg method. The error bars are the standard error calculated for log2 fold change as follows: (std error/mean)∗log2e. Environmental factors were dichotomized as follows: three or more antibiotic treatments across pregnancy and the first year of life, less than four months of exclusive or less than eight months of total breastfeeding, and exposure to fish was defined by consumption more than once per week prenatally or in the first year of life. Positive log2 fold-change of taxa indicates a positive association with future disease or the risk factor.

All but 12 of the 54 most significant taxa higher in ABIS_JIA_ were strongly associated with greater exposure to antibiotics (Fig. 7), while taxa more abundant in controls were associated with the absence of antibiotics. Of the taxa more abundant in controls, *Coprococcus*, *Bifidobacterium breve*, *Bifidobacterium bifidum* were most highly associated with longer durations of breastfeeding. Of those more abundant in ABIS_JIA_, *Acidaminococcales*, *Prevotella 9*, *Bacteroides* sp. (ASV-77), and *Parabacteroides distasonis* (ASV-239) were associated with shorter breastfeeding. *Acidaminococcales* was more abundant in infants with exposure to fish. Taxa that were more abundant in ABIS_JIA_ as well as in children born by CS included *Veillonella parvula* and *Megasphaera micronuciformis*.

## Discussion

It has been hypothesized that microbial aberrations influence JIA disease risk early in childhood.^29^ Our study provides the first evidence, to our knowledge, to demonstrate that as early as one year of age, many years prior to disease onset, microbial differences have already emerged. Environmental early childhood factors increased risk, especially in genetically susceptible children, and were associated with many of the significant taxa.

### Environmental and perinatal factors

There is a sex difference in the prevalence of JIA, with females being more likely to develop JIA than males,^30^^,^^31^ (OR 1.8 [95% CI 1.3–2.6]),^32^ especially oligoarticular and polyarticular JIA,^33^^,^^34^ and in some cases, with an earlier age of onset or higher disease activity.^35^ In ABIS, 66.4% of future JIA (n = 111) were female, compared to 47.7% of controls (n = 16,944), p < 0.001. Age of the mothers at birth was also lower (28 ± 5) in future JIA, compared to controls (30 ± 5), p = 0.008. Evidence on whether maternal age is associated with the risk of JIA in offspring is inconclusive. Although some studies have investigated this, the results have been mixed, with some finding that increased maternal age was associated with increased risk of JIA,^30^^,^^36^ and others with no association.^37^^,^^38^ Birth weight was not associated with JIA risk in ABIS, which is consistent with other studies.^32^^,^^38^ There was no difference in CS between controls and all children with future JIA; however, those delivered by CS were more likely to develop oligoarticular JIA (OR = 2.7; CI 1.3–6.0, p = 0.010). Pooled estimates from a meta-analysis have suggested that CS is a risk factor^38^ while others found no association.^32^

### Microbiome and environmental factors

ABIS_JIA_ were breastfed for a significantly shorter time. Most taxa associated with ABIS_JIA_ had a significant association with shorter durations of breastfeeding, including *Acidaminococcales*, *Prevotella 9*, *Parabacteroides distasonis*, and several ASVs belonging to *Bacteroides*, *Veillonella parvula*, *Turicibacter sanguinis*, *Eggerthella lenta*, and *P. distasonis*. Breast milk secretes IgA and contains mononuclear cells with bacterial DNA, which the infant’s immune system uses to learn to recognize foreign DNA fragments and prepare for future bacterial attacks. Colonization of *Bifidobacterium*, which produces short-chain fatty acids (SCFAs) and maintains intestinal integrity, is promoted by breastfeeding and reduced in children with existing JIA.^18^ Here, several strains were less abundant in ABIS_JIA_. Increased risk of JIA was observed in children exposed to antibiotics early in life. Antibiotics cause significant, but short-term, depletion of bacterial composition and function. Usually, the microbiota recovers within 1–2 months, but exposure to antibiotics early in life can lead to reduced diversity up to two years after exposure.^39^ All taxa associated with ABIS_JIA_ also correlated with early antibiotic exposures. *Prevotella 9* and *Acidaminococcales* were higher in ABIS_JIA_, infants with antibiotic exposures, and infants who had siblings.

### Genetic risk

The association with DR8-DQ4 and ABIS_JIA_ was strong in oligoarthritis. It did not persist when breastfeeding and CS were added to the regression model. Analogous to this, the association between DR1-DQ5 and ABIS_JIA_ was stronger when restricted to those children who had been breastfed for a short time and disappeared when restricted to those breastfed longer. The association between DR8-DQ4 and ABIS_JIA_ (and also oligoarthritis) was stronger only in those who delivered by CS; the association was absent in those who delivered vaginally. DR3-DQ2 and DR15-DQ602 alone were not associated with ABIS_JIA_, but the interaction with exposure to antibiotics showed an increased risk of JIA. This suggests that it is not a risk gene by itself that increases the risk of disease. These gene-environment interactions have not, to our knowledge, been previously studied in JIA.

### Early gut microbiome

Many of the early life microbiome signatures that we observed in ABIS correspond well with extant microbiome studies of children who had already acquired JIA. Unlike other studies in this area, ours is prospective.

Our findings on the microbiota are the first, to our knowledge, to demonstrate that markers of dysbiosis linked to JIA may occur as early as one year of age, with diagnosis at 13.0 ± 5.3 years (Table 1). In ABIS, we have also seen dysbiotic communities in future type-1 diabetes (T1D), with some commonalities to this investigation, including reduced *Fusicatenibacter*, *Phascolarctobacterium*, and *Roseburia* in infants who later acquired T1D^40^; however, many of the most significant bacterial differences in this investigation appear unique to the infants with future JIA. Across the seven studies published to date that have evaluated microbiome differences in children with JIA cross-sectionally, some organisms have consistently emerged, including decreased *Faecalibacterium* and increased *Ruminococcaceae* and *Bacteroides*. A reduced microbial genetic potential to synthesize butyrate,^18^ which is an important substrate for intestinal epithelial cells and can induce the differentiation of regulatory T cells,^41^^,^^42^ has been demonstrated in JIA patients, along with decreased fecal water representation of the butyrate pathway.^43^

Reduced *Anaerostipes*, *Dialister*, *Lachnospira*, and *Roseburia* have been correlated with rheumatic clinical indices including antibodies, erythrocyte sedimentation rate (ESR) and clinical JIA disease activity score.^44^ *Dialister*, *Lachnospira*, and *Roseburia* were three of five genera reduced in ABIS_JIA_, as well as an ASV belonging to *Anaerostipes*. Prevalence was also lower in ABIS_JIA_: *Dialister* (30% in ABIS_JIA_ vs. 64% in controls), *Lachnospira* (30% vs. 63%), *Roseburia* (40% vs. 66%, with *Roseburia hominis* in 0% of ABIS_JIA_ and 25% of controls). These genera produce SCFAs.^45^ That our findings are consistent with the cross-sectional study by Qian et al. is especially interesting, given the geographical and cultural differences (China and Sweden).^44^ *Lachnoclostridium* was less abundant in ABIS_JIA_, as in children with existing JIA.^29^^,^^46^ *Blautia faecis* was completely absent in ABIS_JIA_ but occurred in half of controls; a reduced incidence of *Blautia* has been previously noted cross-sectionally.^17^

ABIS_JIA_ were more likely to lack *Subdoligranulum*, which is an important butyrate-producer and has been shown to be less abundant in enthesitis-related arthritis (ERA) and JIA patients.^15^^,^^17^ A particular strain of *Subdoligranulum* from an individual at risk for rheumatoid arthritis (RA) was recently demonstrated to colonize germ-free mice and generate joint swelling similar to early RA by stimulating complement activation and IgG autoantibodies. However, only one isolate of *Subdoligranulum* (isolate 7 but not isolate 1) was involved.^47^ Observational studies have shown that RA patients have a depletion of anti-inflammatory butyrate-producing bacteria, such as *Fusicatenibacter* and *Subdoligranulum*, in favor of enrichment of pro-inflammatory bacteria, based on a meta-analysis of 92 studies (11,998) with RA.^48^ The gut microbiome of tumor necrosis factor (TNF)-alpha inhibitor (TNFi)-treated patients is characterized by higher abundance of butyrate-producing bacteria such as *Subdoligranulum*, *Lachnospiraceae* ND3007, and *Anaerostipes*, indicating a potential beneficial role for butyrate in the therapeutic effects of TNFi.^49^ Butyrate derived from microbes can suppress the development of autoimmune arthritis by enhancing follicular regulatory T cells that suppress autoantibody production in systemic lymphoid tissue and are negatively associated with disease severity, and improve gut permeability and colonic epithelium health.^50^ *Subdoligranulum*, a known butyrate producer, has also been negatively associated with depressive symptoms in several studies.^51^

Also, an omega-3 rich diet has been shown to result in a significant increase in *Subdoligranulum*, along with other butyrate producers like *Anaerostipes*, *Coprococcus*, and *Roseburia*.^52^ Here, *Anaerostipes* and *Roseburia* were lower in ABIS_JIA_, as well. Omega-3 fatty acids have also been shown to improve active joint counts, swollen joints, TNF-alpha, interluekin-1 (IL-1), and disease activity in children with JIA, reducing the inflammatory response and clinical manifestation.^53^ Low levels of arachidonic acid (AA) and docosahexaenoic acid (DHA) have been described in the active phase of JIA, with total n-3 polyunsaturated fatty acids (PUFAs) lower in JIA and negatively correlated with inflammation.^54^ Many clinical trials have suggested several mechanisms for the effect of omega-3 fatty acids in RA, systemic lupus erythematosus, lupus nephritis, and osteoarthritis. Sixteen out of 20 clinical trials in RA showed significant improvement in disease clinical outcomes.^55^

Here increased *Prevotella 9* prevalence was found in infants who later developed JIA compared to matched controls (30% of ABIS_JIA_ and absent in every matched control). This finding is in contrast to a cross-sectional study of ERA patients, where a reduction in *Prevotellaceae* was observed.^14^ However, an overabundance of *Prevotella copri* in early RA and loss of intestinal barrier integrity (possibly caused by increased bacteria like *Collinsella*) may be responsible for the inflammatory response.^56^ *Prevotella 7* and *Prevotella 9*, the latter of which matched in identity to *P. copri*, have been associated with polygenic risk scores and genetic risk for RA.^57^ In addition, mucosal immune activation and imbalance at the intestinal barrier have been linked to RA pathogenesis.^58^ *Prevotella copri* has been strongly implicated in new-onset untreated RA,^59^^,^^60^ with evidence immune reactivity to the bacterium in a subgroup of these patients.^61^ Potential treatments for RA may target the intestinal barrier integrity to address these underlying factors.^58^ This study further substantiates the possible role of *Prevotella copri* in propagating inflammatory disease and JIA pathogenesis.^60^

Intestinal barrier dysfuction has been described in both JIA and pre-clinical RA. This can promote migration of autoreactive cells to the joints, activation of macrophages, and production of cytokines,^62^ including increased intestinal permeability^12^ and circulating reactivity against bacterial products^63^ in children with JIA. Here, *A muciniphila*, a bacterium shown to have numerous positive effects on intestinal homeostasis, epithelial development, and gut barrier function,^64^ was significantly reduced in infants with future JIA. Although some studies have actually suggested a positive correlation between *A. muciniphila* and arthritis, the results did not reach the threshold for statistical significance.^16^^,^^65^ *Akkermansia muciniphila* can increase short-chain fatty acid levels, suppress pro-inflammatory cytokines, and intriguingly, has been shown to reduce gut damage caused by radiation and methotrexate, a hallmark drug in the treatment of RA and JIA.^64^ In this study, one strain of an *A. muciniphila* was even shown to enhance epithelial regeneration.

*Akkermansia muciniphila* has also been shown to reduce high-fat-diet-induced endotoxemia, strengthen enterocyte monolayer integrity, compete with pathobionts, and repair mucosal damage, without provoking pro-inflammatory reactions.^66^ In pre-clinical RA, it may improve autophagy by enhancing the mucus layer and goblet cell function. *Akkermansia muciniphila* has been shown to reduce mucosal damage from *Salmonella pullorum* in chicks by increasing goblet cells, upregulating mucin 2 (MUC2), and promoting repair through the Wnt/β-catenin pathway, thereby improving colon length, crypt depth, and triggering epithelial proliferation.^67^ Also, viable *A. muciniphila* treatment has been found to reverse high-fat-diet-induced metabolic disorders and increase endocannabinoid levels, which control inflammation and secretion of gut peptides.^68^ Probiotics, prebiotics such as inulin or polysaccharides, and low carbohydrate, ketogenic diets modulate *A. muciniphila*,^69^ which is intriguing since concomitant Celiac Disease, a gluten-related, immune-mediated disorder, has been associated with increased severity of JIA.^70^

Among the most notable ASVs were members of *Bacteroides*, common in previous cross-sectional studies,^13^^,^^14^^,^^16^^,^^18^ including *Bacteroides fragilis* and *Enterococcus* spp.^14^^,^^17^ Increased *Fusobacteria* has been seen in children with the disease,^13^ as well as in ABIS_JIA_ at one year. In ABIS, *Parabacteroides distasonis* in stool at one year was associated with 6.7-increased odds of later contracting JIA (9.0-increased odds after propensity matching). It is speculated that *P. distasonis* fermentation could result in the production of methane, which has been shown to reduce ileal motility and contribute to the pathogenesis of intestinal disorders.^71^ *P. distasonis* is recurrent in Crohn's disease and elevated in ankylosing spondylitis (AS). *In vitro*, this bacterium, along with *Prevotella copri*, increased IFN-gamma-producing cells, possibly a trigger for auto-immunity, and mouse models suggest that *P. distasonis* may be proinflammatory, activating pathogenic toxins and contributing to reactive oxygen species via catalase production.

The small patient sample is a weakness of this study. However, it was expected due to the nature of this general population study, i.e., a birth cohort of a relatively rare disease, and the prevalence of JIA in our study group corresponds well to the prevalence in the general population. As such, complete subgroup analysis of JIA was not achievable. Although there were dropouts, this did not associate with heredity or subsequent diagnosis. No differences were observed between the entire ABIS cohort and those with stool samples; therefore, it seems unlikely that our findings are a result of skewed attrition (e.g., willingness to provide stool or loss to follow-up).

All previous microbiome studies of JIA are cross-sectional, with microbiota acquired after the child has already developed JIA and manifested clinical symptoms. In case-control studies, participants’ outcomes may affect recall of the observed exposure.^72^ A unique feature of the ABIS study is that the microbiome sampling occurred an average of twelve years prior to the JIA diagnosis, which reduces recall bias. Furthermore, here, in ABIS, the link between gut microbiome in infancy and future JIA diagnoses were identified prospectively and hence, have a greater likelihood of being causative.

In conclusion, this is the first investigation to demonstrate that early-life microbiome markers for JIA risk exist prior to disease onset, and furthermore, to provide evidence that they could be harnessed as early as one year of age. Risk factors may be mediated by heredity-environment interactions, as our work demonstrates increased cumulative risk specifically in genetically vulnerable individuals. To better facilitate disease prevention, the mechanistic study into the function of these bacteria and their contribution to the aetiopathogenesis of JIA across time is warranted.

## Contributors

EK and AA performed the formal analysis, prepared the visualizations, and wrote the manuscript. EK and AA contributed equally. Stool samples were processed and microbiome data generated in the laboratory of EWT. JL created and still leads the ABIS study and was responsible for project administration. JL collected all data and biological samples and initiated this study, including funding acquisition. AA curated the microbiome data for this manuscript. EK and AA directly accessed and verified the underlying data reported in the manuscript. EWT and JL supervised this investigation. All authors were involved in conceptualization and methodology. All authors critically reviewed, revised, and approved the manuscript.

## Data sharing statement

Data used in this study are available upon reasonable request from the ABIS director (Johnny Ludvigsson).

## Declaration of interests

We declare no competing interests.

## Appendix A Supplementary data

Supplemental Table S3

Graphical Abstract Risk of juvenile idiopathic arthritis (JIA) may be determined by a combination of gut microbiota, environmental, and genetic factors as early as one year of age.

## References

[1] Meadows S. (2013). https://www.nytimes.com/2013/02/03/magazine/the-boy-with-a-thorn-in-his-joints.html.

[2] Hinks A., Cobb J., Marion M.C. (2013). Dense genotyping of immune-related disease regions identifies 14 new susceptibility loci for juvenile idiopathic arthritis. Nat Genet.

[3] Kristensen K., Henriksen L. (2016). Cesarean section and disease associated with immune function. J Allergy Clin Immunol.

[4] Hyrich K.L., Baildam E., Pickford H. (2016). Influence of past breast feeding on pattern and severity of presentation of juvenile idiopathic arthritis. Arch Dis Child.

[5] Kindgren E., Fredrikson M., Ludvigsson J. (2017). Early feeding and risk of juvenile idiopathic arthritis: a case control study in a prospective birth cohort. Pediatr Rheumatol.

[6] Arvonen M., Virta L.J., Pokka T., Kröger L., Vähäsalo P. (2015). Repeated exposure to antibiotics in infancy: a predisposing factor for juvenile idiopathic arthritis or a sign of this group’s greater susceptibility to infections?. J Rheumatol.

[7] Arvonen M., Berntson L., Pokka T., Karttunen T.J., Vähäsalo P., Stoll M.L. (2016). Gut microbiota-host interactions and juvenile idiopathic arthritis. Pediatr Rheumatol.

[8] Kindgren E., Ludvigsson J. (2021). Infections and antibiotics during fetal life and childhood and their relationship to juvenile idiopathic arthritis: a prospective cohort study. Pediatr Rheumatol.

[9] Kindgren E., Guerrero-Bosagna C., Ludvigsson J. (2019). Heavy metals in fish and its association with autoimmunity in the development of juvenile idiopathic arthritis: a prospective birth cohort study. Pediatr Rheumatol.

[10] Bokulich N.A., Chung J., Battaglia T. (2016). Antibiotics, birth mode, and diet shape microbiome maturation during early life. Sci Transl Med.

[11] Arvonen M., Vähäsalo P., Turunen S. (2012). Altered expression of intestinal human leucocyte antigen D-related and immune signalling molecules in juvenile idiopathic arthritis. Clin Exp Immunol.

[12] Picco P., Gattorno M., Marchese N. (2000). Increased gut permeability in juvenile chronic arthritides. A multivariate analysis of the diagnostic parameters. Clin Exp Rheumatol.

[13] Tejesvi M.V., Arvonen M., Kangas S.M. (2016). Faecal microbiome in new-onset juvenile idiopathic arthritis. Eur J Clin Microbiol Infect Dis.

[14] Aggarwal A., Sarangi A.N., Gaur P., Shukla A., Aggarwal R. (2017). Gut microbiome in children with enthesitis-related arthritis in a developing country and the effect of probiotic administration. Clin Exp Immunol.

[15] Di Paola M., Cavalieri D., Albanese D. (2016). Alteration of fecal microbiota profiles in juvenile idiopathic arthritis. Associations with HLA-B27 allele and disease status. Front Microbiol.

[16] Stoll M.L., Kumar R., Morrow C.D. (2014). Altered microbiota associated with abnormal humoral immune responses to commensal organisms in enthesitis-related arthritis. Arthritis Res Ther.

[17] van Dijkhuizen E.H.P., Del Chierico F., Malattia C. (2019). Microbiome analytics of the gut microbiota in patients with juvenile idiopathic arthritis: a longitudinal observational cohort study. Arthritis Rheumatol.

[18] Stoll M.L., Weiss P.F., Weiss J.E. (2018). Age and fecal microbial strain-specific differences in patients with spondyloarthritis. Arthritis Res Ther.

[19] Dong Y.-Q., Wang W., Li J. (2019). Characterization of microbiota in systemic-onset juvenile idiopathic arthritis with different disease severities. World J Clin Cases.

[20] Ilonen J., Kiviniemi M., Lempainen J. (2016). Genetic susceptibility to type 1 diabetes in childhood - estimation of HLA class II associated disease risk and class II effect in various phases of islet autoimmunity. Pediatr Diabetes.

[21] Caporaso J.G., Kuczynski J., Stombaugh J. (2010). QIIME allows analysis of high-throughput community sequencing data. Nat Methods.

[22] Callahan B.J., McMurdie P.J., Rosen M.J., Han A.W., Johnson A.J.A., Holmes S.P. (2016). DADA2: high-resolution sample inference from Illumina amplicon data. Nat Methods.

[23] Quast C., Pruesse E., Yilmaz P. (2013). The SILVA ribosomal RNA gene database project: improved data processing and web-based tools. Nucleic Acids Res.

[24] (2023). Microbiome R package. https://github.com/microbiome/microbiome.

[25] Oksanen J., Simpson G.L., Blanchet F.G. (2022). vegan: community ecology package. https://CRAN.R-project.org/package=vegan.

[26] Love M.I., Huber W., Anders S. (2014). Moderated estimation of fold change and dispersion for RNA-seq data with DESeq2. Genome Biol.

[27] Ho D., Imai K., King G., Stuart E.A. (2011). MatchIt: nonparametric preprocessing for parametric causal inference. J Stat Softw.

[28] Roesch L.F.W., Dobbler P.T., Pylro V.S., Kolaczkowski B., Drew J.C., Triplett E.W. (2020). pime: a package for discovery of novel differences among microbial communities. Mol Ecol Resour.

[29] Arvonen M., Vänni P., Sarangi A.N. (2020). Microbial orchestra in juvenile idiopathic arthritis: sounds of disarray?. Immunol Rev.

[30] Bell S.W., Shenoi S., Nelson J.L., Bhatti P., Mueller B.A. (2017). Juvenile idiopathic arthritis in relation to perinatal and maternal characteristics: a case control study. Pediatr Rheumatol Online J.

[31] Mannion M.L., Xie F., Beukelman T., Investigators for the CR (2022). Investigation of inactive disease states among patients with juvenile idiopathic arthritis in the Childhood Arthritis and Rheumatology Research Alliance registry. ACR Open Rheumatol.

[32] Shenoi S., Shaffer M., Wallace C. (2016). Environmental risk factors and early life exposures for juvenile idiopathic arthritis; a case: control study. Arthritis Care Res (Hoboken).

[33] Zaripova L.N., Midgley A., Christmas S.E., Beresford M.W., Baildam E.M., Oldershaw R.A. (2021). Juvenile idiopathic arthritis: from aetiopathogenesis to therapeutic approaches. Pediatr Rheumatol.

[34] Barut K., Adrovic A., Şahin S., Kasapçopur Ö. (2017). Juvenile idiopathic arthritis. Balkan Med J.

[35] Cattalini M., Soliani M., Caparello M.C., Cimaz R. (2019). Sex differences in pediatric rheumatology. Clin Rev Allergy Immunol.

[36] Shenoi S., Bell S., Wallace C.A., Mueller B.A. (2015). Juvenile idiopathic arthritis in relation to maternal prenatal smoking. Arthritis Care Res (Hoboken).

[37] Carlens C., Jacobsson L., Brandt L., Cnattingius S., Stephansson O., Askling J. (2009). Perinatal characteristics, early life infections and later risk of rheumatoid arthritis and juvenile idiopathic arthritis. Ann Rheum Dis.

[38] Clarke S.L.N., Mageean K.S., Maccora I. (2022). Moving from nature to nurture: a systematic review and meta-analysis of environmental factors associated with juvenile idiopathic arthritis. Rheumatology.

[39] Tapiainen T., Koivusaari P., Brinkac L. (2019). Impact of intrapartum and postnatal antibiotics on the gut microbiome and emergence of antimicrobial resistance in infants. Sci Rep.

[40] Bélteky M., Milletich P.L., Ahrens A.P., Triplett E.W., Ludvigsson J. (2023). Infant gut microbiome composition correlated with type 1 diabetes acquisition in the general population: the ABIS study. Diabetologia.

[41] Hamer H.M., Jonkers D., Venema K., Vanhoutvin S., Troost F.J., Brummer R.-J. (2008). Review article: the role of butyrate on colonic function. Aliment Pharmacol Ther.

[42] Smith P.M., Howitt M.R., Panikov N. (2013). The microbial metabolites, short-chain fatty acids, regulate colonic Treg cell homeostasis. Science.

[43] Stoll M.L., Kumar R., Lefkowitz E.J., Cron R.Q., Morrow C.D., Barnes S. (2016). Fecal metabolomics in pediatric spondyloarthritis implicate decreased metabolic diversity and altered tryptophan metabolism as pathogenic factors. Genes Immun.

[44] Qian X., Liu Y.-X., Ye X. (2020). Gut microbiota in children with juvenile idiopathic arthritis: characteristics, biomarker identification, and usefulness in clinical prediction. BMC Genomics.

[45] Tamanai-Shacoori Z., Smida I., Bousarghin L. (2017). *Roseburia* spp.: a marker of health?. Future Microbiol.

[46] Öman A., Dicksved J., Engstrand L., Berntson L. (2021). Fecal microbiota in children with juvenile idiopathic arthritis treated with methotrexate or etanercept. Pediatr Rheumatol.

[47] Chriswell M.E., Lefferts A.R., Clay M.R. (2022). Clonal IgA and IgG autoantibodies from individuals at risk for rheumatoid arthritis identify an arthritogenic strain of *Subdoligranulum*. Sci Transl Med.

[48] Wang Y., Wei J., Zhang W. (2022). Gut dysbiosis in rheumatic diseases: a systematic review and meta-analysis of 92 observational studies. eBioMedicine.

[49] Koh J.H., Lee E.H., Cha K.H., Pan C.-H., Kim D., Kim W.-U. (2023). Factors associated with the composition of the gut microbiome in patients with established rheumatoid arthritis and its value for predicting treatment responses. Arthritis Res Ther.

[50] Takahashi D., Hoshina N., Kabumoto Y. (2020). Microbiota-derived butyrate limits the autoimmune response by promoting the differentiation of follicular regulatory T cells. EBioMedicine.

[51] Radjabzadeh D., Bosch J.A., Uitterlinden A.G. (2022). Gut microbiome-wide association study of depressive symptoms. Nat Commun.

[52] Noriega B.S., Sanchez-Gonzalez M.A., Salyakina D., Coffman J. (2016). Understanding the impact of omega-3 rich diet on the gut microbiota. Case Rep Med.

[53] Gheita T., Kamel S., Helmy N., El-Laithy N., Monir A. (2012). Omega-3 fatty acids in juvenile idiopathic arthritis: effect on cytokines (IL-1 and TNF-α), disease activity and response criteria. Clin Rheumatol.

[54] Gorczyca D., Postępski J., Czajkowska A. (2017). The profile of polyunsaturated fatty acids in juvenile idiopathic arthritis and association with disease activity. Clin Rheumatol.

[55] Akbar U., Yang M., Kurian D., Mohan C. (2017). Omega-3 fatty acids in rheumatic diseases: a critical review. J Clin Rheumatol.

[56] Tsetseri M.-N., Silman A.J., Keene D.J., Dakin S.G. (2023). The role of the microbiome in rheumatoid arthritis: a review. Rheumatol Adv Pract.

[57] Wells P.M., Adebayo A.S., Bowyer R.C.E. (2020). Associations between gut microbiota and genetic risk for rheumatoid arthritis in the absence of disease: a cross-sectional study. Lancet Rheumatol.

[58] Brandl C., Bucci L., Schett G., Zaiss M.M. (2021). Crossing the barriers: revisiting the gut feeling in rheumatoid arthritis. Eur J Immunol.

[59] Scher J.U., Sczesnak A., Longman R.S. (2013). Expansion of intestinal *Prevotella copri* correlates with enhanced susceptibility to arthritis. Elife.

[60] Stoll M.L. (2020). Genetics, *Prevotella*, and the pathogenesis of rheumatoid arthritis. Lancet Rheumatol.

[61] Pianta A., Arvikar S., Strle K. (2017). Evidence of the immune relevance of *Prevotella copri*, a gut microbe, in patients with rheumatoid arthritis. Arthritis Rheumatol.

[62] Zhao T., Wei Y., Zhu Y. (2022). Gut microbiota and rheumatoid arthritis: from pathogenesis to novel therapeutic opportunities. Front Immunol.

[63] Fotis L., Shaikh N., Baszis K.W. (2017). Serologic evidence of gut-driven systemic inflammation in juvenile idiopathic arthritis. J Rheumatol.

[64] Kim S., Shin Y.-C., Kim T.-Y. (2021). Mucin degrader *Akkermansia muciniphila* accelerates intestinal stem cell-mediated epithelial development. Gut Microb.

[65] Stoll M.L., Pierce M.K., Watkins J.A. (2019). Akkermansia muciniphila is permissive to arthritis in the K/BxN mouse model of arthritis. Genes Immun.

[66] Reunanen J., Kainulainen V., Huuskonen L. (2015). *Akkermansia muciniphila* adheres to enterocytes and strengthens the integrity of the epithelial cell layer. Appl Environ Microbiol.

[67] Zhu L., Lu X., Liu L., Voglmeir J., Zhong X., Yu Q. (2020). *Akkermansia muciniphila* protects intestinal mucosa from damage caused by *S. pullorum* by initiating proliferation of intestinal epithelium. Vet Res.

[68] Everard A., Belzer C., Geurts L. (2013). Cross-talk between *Akkermansia muciniphila* and intestinal epithelium controls diet-induced obesity. Proc Natl Acad Sci U S A.

[69] Hagi T., Belzer C. (2021). The interaction of *Akkermansia muciniphila* with host-derived substances, bacteria and diets. Appl Microbiol Biotechnol.

[70] Naddei R., Di Gennaro S., Guarino A., Troncone R., Alessio M., Discepolo V. (2022). In a large juvenile idiopathic arthritis (JIA) cohort, concomitant celiac disease is associated with family history of autoimmunity and a more severe JIA course: a retrospective study. Pediatr Rheumatol.

[71] Ezeji J.C., Sarikonda D.K., Hopperton A. (2021). *Parabacteroides distasonis*: intriguing aerotolerant gut anaerobe with emerging antimicrobial resistance and pathogenic and probiotic roles in human health. Gut Microb.

[72] Gilbert R., Martin R.M., Donovan J. (2016). Misclassification of outcome in case-control studies: methods for sensitivity analysis. Stat Methods Med Res.

