# Supplementary material for: Infant gut microbiota and environment associate with juvenile idiopathic arthritis many years prior to disease onset, especially in genetically vulnerable children
Source: eBioMedicine. 2023 Jun 15;93:104654. doi: 10.1016/j.ebiom.2023.104654 (PMC10279551; doi:10.1016/j.ebiom.2023.104654)
Supplement: Supplemental Table S2 [file mmc2.docx]

**Supplemental Table 2. Cohort characteristics between Controls and future JIA groups before and after propensity score matching (PSM).** Data are n (%) or mean (standard deviation, SD), with p values from t-tests or χ^2^. Comparisons were run between the control and JIA groups before matching and after matching, separately. Data were derived from the birth questionnaire or one-year questionnaire. Medication types during pregnancy included antibiotics, corticosteroids, hypertensive medications, psychiatric medications, pain killers, hormone preparates, and cytostatics. PSM = propensity score matching.

|  | Full Microbiome Cohort | | | Post-PSM | | |
| --- | --- | --- | --- | --- | --- | --- |
|  | Control | JIA | p | Control | JIA | p |
| n | 1671 | 12 |  | 36 | 12 |  |
| Gestational age, weeks (mean (SD)) | 39.73 (1.80) | 39.67 (1.07) | 0.905 | 39.94 (1.57) | 39.67 (1.07) | 0.572 |
| Biological sex, Female (%) | 797 (48.1) | 8 (66.7) | 0.322 | 20 (55.6) | 8 (66.7) | 0.735 |
| Mode of delivery, C-section (%) | 175 (11.7) | 2 (16.7) | 0.935 | 1 (2.8) | 2 (16.7) | 0.302 |
| Use of antibiotics during pregnancy, No (%) | 1183 (78.0) | 8 (66.7) | 0.554 | 30 (88.2) | 8 (66.7) | 0.211 |
| Region of Sweden (%) |  |  | 0.451 |  |  | 0.946 |
|  | 19 (1.1) | 0 (0.0) |  |  |  |  |
| Central Region | 456 (27.3) | 3 (25.0) |  | 10 (27.8) | 3 (25.0) |  |
| Northern Region | 495 (29.6) | 6 (50.0) |  | 16 (44.4) | 6 (50.0) |  |
| Southern Region | 701 (42.0) | 3 (25.0) |  | 10 (27.8) | 3 (25.0) |  |
| Municipality |  |  | 0.89 |  |  | 0.068 |
| DR1-DQ5 (%) | 322 (23.2) | 7 (58.3) | 0.012 | 21 (58.3) | 7 (58.3) | 1 |
| Infection during pregnancy, No (%) | 1069 (69.1) | 7 (63.6) | 0.949 | 27 (81.8) | 7 (63.6) | 0.406 |
| Exclusive breastfeeding duration, months (%) |  |  | 0.295 |  |  | 0.798 |
| 1 | 154 (11.1) | 1 (12.5) |  | 5 (16.1) | 1 (12.5) |  |
| 2 | 65 (4.7) | 2 (25.0) |  | 2 (6.5) | 2 (25.0) |  |
| 3 | 92 (6.6) | 1 (12.5) |  | 3 (9.7) | 1 (12.5) |  |
| 4 | 462 (33.2) | 3 (37.5) |  | 12 (38.7) | 3 (37.5) |  |
| 5 | 233 (16.8) | 0 (0.0) |  | 2 (6.5) | 0 (0.0) |  |
| 6 | 248 (17.8) | 1 (12.5) |  | 3 (9.7) | 1 (12.5) |  |
| 7 | 62 (4.5) | 0 (0.0) |  | 3 (9.7) | 0 (0.0) |  |
| 8 | 24 (1.7) | 0 (0.0) |  |  |  |  |
| 9 | 51 (3.7) | 0 (0.0) |  | 1 (3.2) | 0 (0.0) |  |
| Total breastfeeding duration, months (%) |  |  | 0.089 |  |  | 0.062 |
| 1 | 64 (4.5) | 1 (11.1) |  | 3 (9.7) | 1 (11.1) |  |
| 2 | 56 (3.9) | 2 (22.2) |  | 0 (0.0) | 2 (22.2) |  |
| 3 | 60 (4.2) | 0 (0.0) |  | 1 (3.2) | 0 (0.0) |  |
| 4 | 61 (4.3) | 0 (0.0) |  | 3 (9.7) | 0 (0.0) |  |
| 5 | 65 (4.6) | 1 (11.1) |  | 0 (0.0) | 1 (11.1) |  |
| 6 | 138 (9.7) | 2 (22.2) |  | 3 (9.7) | 2 (22.2) |  |
| 7 | 154 (10.8) | 0 (0.0) |  | 3 (9.7) | 0 (0.0) |  |
| 8 | 209 (14.7) | 0 (0.0) |  | 5 (16.1) | 0 (0.0) |  |
| 9 | 613 (43.2) | 3 (33.3) |  | 13 (41.9) | 3 (33.3) |  |
| Infection with antibiotics in the first 12 months of life (%) |  |  | <0.001 |  |  | 0.111 |
| Never | 801 (62.6) | 7 (87.5) |  | 21 (72.4) | 7 (87.5) |  |
| 1-2 times | 410 (32.0) | 0 (0.0) |  | 7 (24.1) | 0 (0.0) |  |
| 3-5 times | 65 (5.1) | 0 (0.0) |  | 1 (3.4) | 0 (0.0) |  |
| More than 5 times | 4 (0.3) | 1 (12.5) |  | 0 (0.0) | 1 (12.5) |  |
| Infection as newborn, Yes (%) | 66 (4.7) | 2 (22.2) | 0.096 | 1 (3.3) | 2 (22.2) | 0.249 |
| Total number of medication types during pregnancy (%) |  |  | 0.004 |  |  | 0.053 |
| 0 medications | 502 (36.9) | 2 (20.0) |  | 7 (22.6) | 2 (20.0) |  |
| 1 | 506 (37.2) | 2 (20.0) |  | 16 (51.6) | 2 (20.0) |  |
| 2 | 264 (19.4) | 5 (50.0) |  | 5 (16.1) | 5 (50.0) |  |
| 3 (93rd percentile) | 78 (5.7) | 0 (0.0) |  | 3 (9.7) | 0 (0.0) |  |
| 4 | 12 (0.9) | 1 (10.0) |  | 0 (0.0) | 1 (10.0) |  |
