# Supplementary material for: Infant gut microbiota and environment associate with juvenile idiopathic arthritis many years prior to disease onset, especially in genetically vulnerable children
Source: eBioMedicine. 2023 Jun 15;93:104654. doi: 10.1016/j.ebiom.2023.104654 (PMC10279551; doi:10.1016/j.ebiom.2023.104654)
Supplement: Supplemental Table S3 [file mmc3.docx]

**Supplemental Table 3. Confounds of microbial diversity in ABIS.** PERMANOVA statistics for the microbial diversity index at the amplicon sequence variant (ASV)-level using the Bray-Curtis method with 1000 permutations. Abundances underwent a compositional transformation using the microbiome R package, and then PERMANOVAs were calculated using the adonis function of the vegan R package. In Model 1, terms were added sequentially. Human leukocyte antigen (HLA) haplotypes were entered as binary features: 0 (absence of the allele), 1 (heterozygous or homozygous for the allele). For Models 2-5, continuous body mass index (BMI) was assessed separately at each age due to missing values across time. Significance codes: p<0.001 (***), p<0.01 (**), p<0.05 (*).

| Model (n) | *df* | *F model* | *R^2^* | *p* |
| --- | --- | --- | --- | --- |
| **Model 1 (n=1387)** |  |  |  |  |
| Biological sex | 1 | 1.75361 | 0.00125 | 0.005994 ** |
| County | 6 | 2.66032 | 0.01140 | 0.000999 *** |
| DR1-DQ5 | 1 | 1.17807 | 0.00084 | 0.204795 |
| DR13-DQ603 | 1 | 1.36390 | 0.00097 | 0.060939 |
| DR13-DQ604 | 1 | 0.84719 | 0.00060 | 0.726274 |
| DR14-DQ5 | 1 | 1.38097 | 0.00099 | 0.068931 |
| DR14-DQ503 | 1 | 1.11243 | 0.00079 | 0.276723 |
| DR15-DQ601 | 1 | 0.95304 | 0.00068 | 0.543457 |
| DR15-DQ602 | 1 | 0.88866 | 0.00063 | 0.633367 |
| DR16-DQ5 | 1 | 0.85851 | 0.00061 | 0.716284 |
| DR16-DQ502 | 1 | 0.77844 | 0.00056 | 0.860140 |
| DR3-DQ2.5 | 1 | 1.22696 | 0.00088 | 0.142857 |
| DR4-DQ7 | 1 | 1.32368 | 0.00095 | 0.095904 |
| DR4-DQ8 | 1 | 0.91544 | 0.00065 | 0.603397 |
| DR5-DQ7 | 1 | 0.91497 | 0.00065 | 0.602398 |
| DR7-DQ2 | 1 | 1.23308 | 0.00088 | 0.160839 |
| DR7-DQ9 | 1 | 0.97196 | 0.00069 | 0.482517 |
| DR8-DQ4 | 1 | 0.73032 | 0.00052 | 0.904096 |
| DR9-DQ9 | 1 | 1.05206 | 0.00075 | 0.371628 |
| **Models 2-5** |  |  |  |  |
| BMI at one year (n=1315) | 1 | 0.8728 | 0.00066 | 0.6843 |
| BMI at two years (n=59) | 1 | 1.1399 | 0.01961 | 0.2328 |
| BMI at five years (n=65) | 1 | 1.0784 | 0.01683 | 0.3187 |
| BMI at eight years (n=27) | 1 | 1.0935 | 0.04191 | 0.3017 |
