# Supplementary material for: Infant gut microbiota and environment associate with juvenile idiopathic arthritis many years prior to disease onset, especially in genetically vulnerable children
Source: eBioMedicine. 2023 Jun 15;93:104654. doi: 10.1016/j.ebiom.2023.104654 (PMC10279551; doi:10.1016/j.ebiom.2023.104654)
Supplement: Graphical Abstract — Risk of juvenile idiopathic arthritis (JIA) may be determined by a combination of gut microbiota, environmental, and genetic factors as early as one year of age. [file mmc4.pdf]

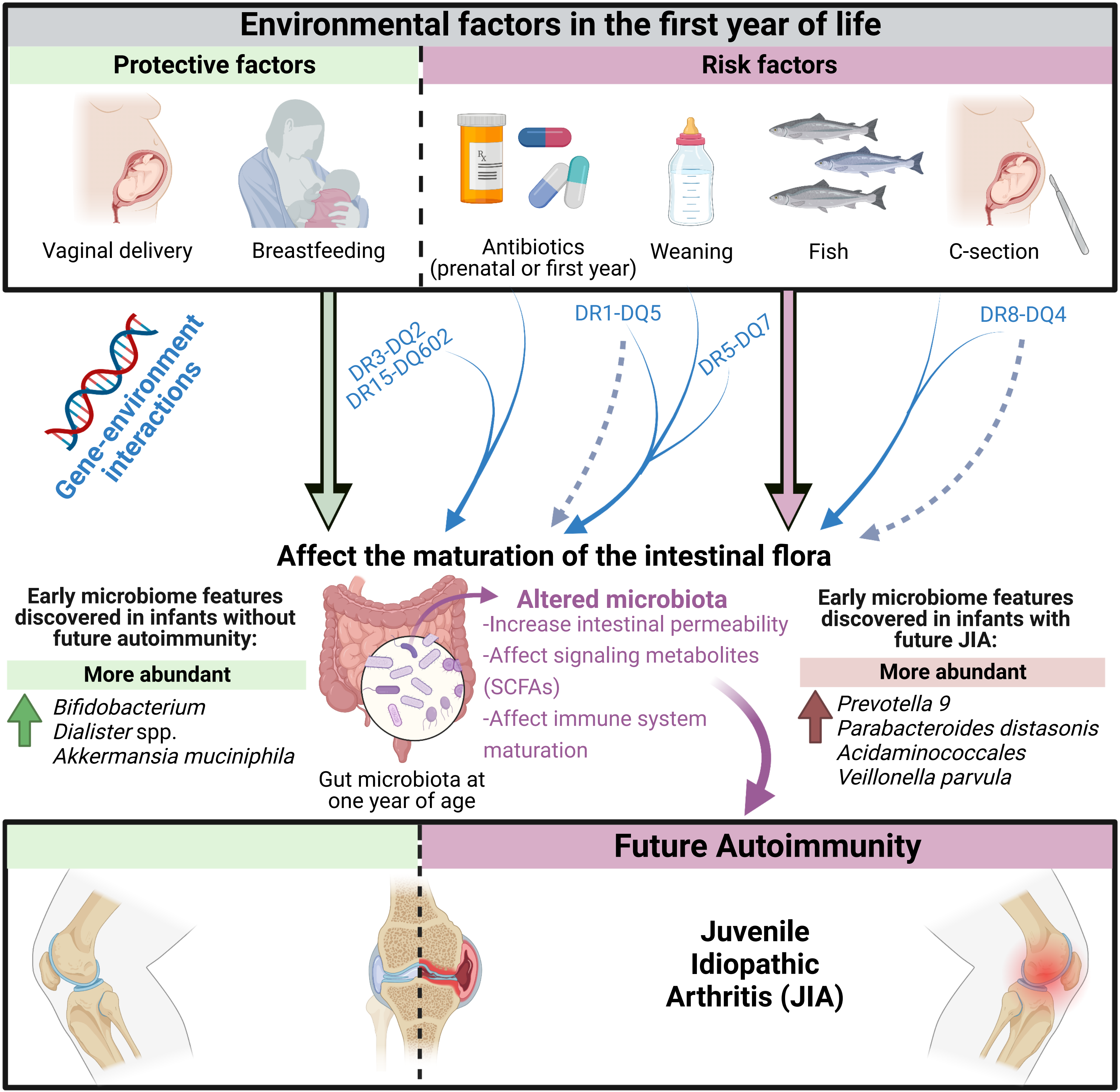
Environmental factors in the first year of life
Protective factors
Risk factors
Vaginal delivery
Breastfeeding
Antibiotics (prenatal or first year)
Weaning
Fish
C-section
Gene-environment interactions
DR3-DQ2
DR15-DQ602
DR1-DQ5
DR5-DQ7
DR8-DQ4
Affect the maturation of the intestinal flora
Early microbiome features discovered in infants without future autoimmunity:
More abundant
Bifidobacterium
Dialister spp.
Akkermansia muciniphila
Gut microbiota at one year of age
Altered microbiota
-Increase intestinal permeability
-Affect signaling metabolites (SCFAs)
-Affect immune system maturation
Early microbiome features discovered in infants with future JIA:
More abundant
Prevotella 9
Parabacteroides distasonis
Acidaminococcales
Veillonella parvula
Future Autoimmunity
Juvenile Idiopathic Arthritis (JIA)
